# Differential hypothalamic leptin sensitivity in obese rat offspring exposed to maternal and postnatal intake of chocolate and soft drink

**DOI:** 10.1038/nutd.2016.53

**Published:** 2017-01-16

**Authors:** M Kjaergaard, C Nilsson, A Secher, J Kildegaard, T Skovgaard, M O Nielsen, K Grove, K Raun

**Affiliations:** 1 Diabetes and Obesity Pharmacology, Novo Nordisk A/S, Måløv, Denmark; 2 Department of Large Animal Science, Faculty of Health and Medical Sciences, University of Copenhagen, Frederiksberg C, Denmark; 3 Uppsala University Innovation, Uppsala Science Park, Uppsala, Sweden; 4 Division of Diabetes, Obesity and Metabolism, Oregon National Primate Research Center, Oregon Health and Science University, Beaverton, OR, USA

## Abstract

**Background/objective:**

Intake of high-energy foods and maternal nutrient overload increases the risk of metabolic diseases in the progeny such as obesity and diabetes. We hypothesized that maternal and postnatal intake of chocolate and soft drink will affect leptin sensitivity and hypothalamic astrocyte morphology in adult rat offspring.

**Methods:**

Pregnant Sprague-Dawley rats were fed *ad libitum* chow diet only (C) or with chocolate and high sucrose soft drink supplement (S). At birth, litter size was adjusted into 10 male offspring per mother. After weaning, offspring from both dietary groups were assigned to either S or C diet, giving four groups until the end of the experiment at 26 weeks of age.

**Results:**

As expected, adult offspring fed the S diet post weaning became obese (body weight: *P*<0.01, %body fat per kg: *P*<0.001) and this was due to the reduced energy expenditure (*P*<0.05) and hypothalamic astrogliosis (*P*<0.001) irrespective of maternal diet. Interesting, offspring born to S-diet-fed mothers and fed the S diet throughout postnatal life became obese despite lower energy intake than controls (*P*<0.05). These SS offspring showed increased feed efficiency (*P*<0.001) and reduced fasting pSTAT3 activity (*P*<0.05) in arcuate nucleus (ARC) compared with other groups. The findings indicated that the combination of the maternal and postnatal S-diet exposure induced persistent changes in leptin signalling, hence affecting energy balance. Thus, appetite regulation was more sensitive to the effect of leptin than energy expenditure, suggesting differential programming of leptin sensitivity in ARC in SS offspring. Effects of the maternal S diet were normalized when offspring were fed a chow diet after weaning.

**Conclusions:**

Maternal intake of chocolate and soft drink had long-term consequences for the metabolic phenotype in the offspring if they continued on the S diet in postnatal life. These offspring displayed obesity despite lowered energy intake associated with alterations in hypothalamic leptin signalling.

## Introduction

The hypothalamus is a prominent brain area involved in the control energy homoeostasis.^1, 2, 3^ Within the hypothalamus, neurons in the arcuate nucleus (ARC) integrate information from other regions of the brain, as well as hormonal and nutrient signals from the periphery. Leptin, an adipocyte expressed hormone, influences the hypothalamic regulation of energy homoeostasis to suppress food intake and increase energy expenditure. Although hypothalamic resistance to leptin results in hyperphagia and reduced energy expenditure leading to obesity, the exact mechanism by which leptin resistance develops is not completely clarified.^1, 2, 3^ Neuronal inflammation within the hypothalamus has been proposed as one mechanism leading to hypothalamic leptin resistance.^4, 5, 6^ Recently, astrocytes have been proposed to be involved in hypothalamic leptin signalling.^5, 7, 8^ They are fundamental for normal brain development and function, as they mediate neuronal proliferation, survival and metabolism.^9, 10, 11^ It was reported that within few days of high-fat-diet (HFD) feeding, astrocytes entered into a reactive state as an initiated transient neuroprotective effect.^7, 12^ This is termed astrogliosis and characterized by increased proliferation, changed morphology and increased expression of glial fibrillary acidic protein (GFAP).^11, 13^ Long-term astrogliosis has on the other hand been associated with hypothalamic neuronal damage, inflammation and impairment of leptin signalling leading to obesity^7, 14^ but the underlying mechanisms are not understood. It has been shown that astrocytes contain leptin receptors; thus, astrogliosis has been proposed to induce a barrier for leptin to reach neurons and exert its control on energy balance.^5, 6^

Predisposition to obesity and type 2 diabetes can arise during foetal development and in early postnatal life.^15, 16, 17^ This is asserted to imbalances in maternal nutritional, hormonal and external environment that can alter neuronal circuits of the brain^18, 19, 20, 21^ as well as other essential organs^22^ that are critical to regulation of energy homoeostasis.^16, 17^ For instance, a maternal HFD induces changes in hormone levels such as leptin, insulin and ghrelin.^18, 23, 24, 25^ Changes of these hormones in offspring during early postnatal life can potentially disrupt hypothalamic neuronal formation,^18, 26, 27^ which is essential for leptin's regulation of energy homoeostasis.^26, 28^

In the present study, we hypothesized that maternal and/or postnatal chocolate and soft drink supplement (S) will affect hypothalamic leptin signalling and astrocyte morphology, predisposing to offspring to obesity. As a measure of neuronal damage, we determined hypothalamic astrocyte accumulation via expression of GFAP. Leptin sensitivity was determined by measuring acute food intake in response to an intraperitoneal injection of leptin and through determination of fasting hypothalamic STAT3 phosphorylation. Furthermore, we investigated whether a postnatal chow diet after weaning could rescue the adverse effects of exposure to the maternal S diet.

## Materials and methods

### Experimental animals

The experimental procedures in the present study were approved by the Animal Experiments Inspectorate (2005/561-989), under Danish Ministry of Justice, and are in accordance with the Danish Animal Experimentations Act. The study was performed at the Laboratory Animal Unit at Novo Nordisk A/S, Maaloev, Denmark.

### Study design

**Figure 1:**
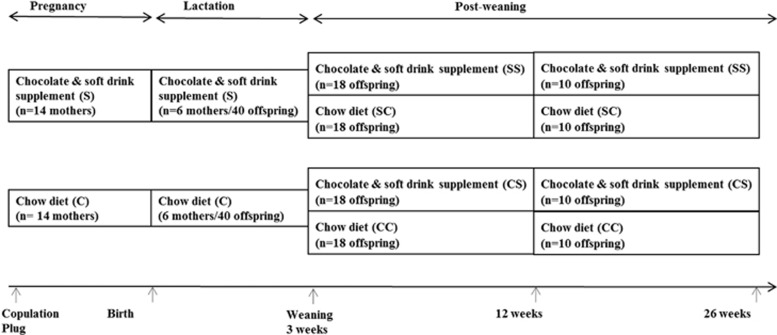
The rat study design. Twenty-eight pregnant Sprague-Dawley rats were either fed *ad libitum* chow diet only (C) or chow with chocolate and high sucrose soft drink supplement (S) from 1 day after copulation plug and until weaning of offspring 3 weeks after parturition. At birth, a representative number of male offspring (*n*=112) were selected and used for analyses. Litter size was adjusted into 10 male offspring per mother. After weaning at 3 weeks of age, offspring from both dietary groups were assigned to either S or C diet, giving four groups until the end of the experiment at 26 weeks of age, namely CC, CS, SC and SS. For details of animals and procedures, see Experimental animals in Materials and Methods.

Twenty-eight pregnant Spraque-Dawley (SD) rats, aged nine weeks with body weight ranging from 200 to 250 g, were used in the experiments. The mating took place at the Taconic animal facilities (Taconic Europe, Lille Skensved, Denmark), and the pregnant rats were transported to the Laboratory Animal Unit at Novo Nordisk A/S, Måløv, Denmark, at day 1 after confirmation of gestational plug. The SD males used for mating were fed a standard laboratory chow diet (Altromin 1320, Brogaarden, Denmark), and were of normal body weight. The pregnant rats were housed individually in cages on a light–dark cycle 12:12 at a temperature of 20 ºC. All rats had free access to water from drinking nipples. The diet intervention of the pregnant rats was initiated one day after ensuring gestational plug. The pregnant rats were randomly assigned to one of two experimental diets (Figure 1); one group (*n*=14) was assigned to a chow diet supplemented *ad libitum* with chocolate and soft drink supplement (S), and the other group (*n*=14) received the chow (C) diet only. At birth, male offspring (*n*=112) were selected for the study and used for analysis at different developmental time points. Thereafter surplus offspring were killed. For fostering purposes in order to ensure sufficient milk intake, we selected mothers having normal litter sizes (*n*=10–14). In each dietary group (C and S), male offspring were randomly cross-fostered into litter size of 10 offspring per mother. At weaning (3 weeks of age), offspring were assigned to either the S or C diets until the end of the experiment at 26 weeks of age, giving rise to four dietary groups; SS, SC, CS and CC (Figure 1). The first letter refers to the maternal diet (pregnancy and lactation), second letter to the diet from weaning to 26 weeks of age. Blood samples and tissues were collected from the offspring after their killing at different developmental stages, at day 1 (*n*=10 per group) as well as at week 3 (*n*=10 per group), week 12 (*n*=8 per group) and 26 weeks of age (*n*=10 per group).

### Diets and food intake

All rats had free access to water from drinking nipples and were fed a standard laboratory chow diet *ad libitum* (Altromin 1320). The chow diet contained a caloric density of 2.8 kcal g^−1^ (24% protein, 11% fat and 65% carbohydrates). The high-fat/high-sucrose supplementary diet consisted of various chocolate bars on an average caloric density of ~5.4 kcal g^−1^ (8% protein, 33% fat, 59% carbohydrates of which 53% sucrose). The soft drink was produced according to the European standards. It consisted of a caloric density of on average 1.96 kcal ml^−1^ (0% protein, 0% fat, 100% sucrose), and was given *ad libitum* in bottles (at least 80 ml per day) and changed every third day. Chocolate and soft drink intake was recorded by manual weighing once a week. The maternal intake of chocolate and soft drink was estimated to be ~40 kcal per week during gestation and 50 kcal per week during lactation. Thus, the intake of chocolate and soft drink was 20 and 15–20% out of the total caloric intake, respectively (see Kjaergaard *et al.*^29^ for further details). For each offspring, chow and chocolate intake was recorded by manual weighing once a day during 5 days at 11 and 24 weeks of age. The energy intake was calculated in kcal ingested daily per rat and presented as daily energy consumption per group.

### Body composition

Body composition was determined by the Echo Medical System per rat QMR scanner (EchoMRI 2004, Houston, TX, USA) at 12 and 25 weeks of age, as previously described.^29^

### Energy expenditure

Energy expenditure was determined at 12 and 26 weeks of age by indirect calorimetry as previously described.^30^ Data were collected for 22 h (10 h light and 12 h dark).

### Blood analysis

Blood samples used for glucose, insulin, TG and FFA determination were analysed as previously described.^29^ Blood for leptin was sampled in EDTE-coated tubes and plasma (30 μl) and was analysed by AlphaLISA mouse leptin kit from AlphaLISA Research Reagents (product no.: AL521 C/F, lot no.: 1696861, PerkinElmer BioSignal Inc, Waltham, MA, USA).

### RNA extraction and real-time PCR

Cellular RNA and PCR reactions were performed on the whole rat hypothalamus at 3 and 12 weeks of age as previously described.^29^ The target genes were NPY (Rn00561681_m1), AGRP (Rn01431702_g1), POMC (Mm00435874_m1), GALp (Rn00575275_m1), MC4R (Mm00457483_s1), CART (Mm00489086_m1), 18 S ribosomal RNA (Mm04277571_s1) (AppliedBiosystems, Roskilde, Denmark). Levels of expression of each target gene of interest were presented as a percentage of the expression of the appropriate housekeeping gene 18 S ribosomal RNA.

### Leptin responsiveness

Leptin responsiveness was assessed by measuring food intake in response to intraperitoneal (i.p.) saline or leptin injection in offspring aged 24 weeks. Offspring were semi-fasted overnight, only receiving 15 g of food from 1430 to 0600 hours the following day. From 0600 to 1800 hours, the offspring were completely fasted, only allowing water intake. Offspring were i.p. injected with saline or leptin (~4.0 mg kg^−1^). After 4, 15 and 24 h, food intake was recorded.

Fasting central leptin responsiveness was assessed by quantifying phosphorylated signal transducer and activator of transcription-3 (pSTAT3)-immunoreactive cells in offspring aged 26 weeks. Brain extraction was performed as previously described,^31^ only modified by using borate-buffered 4% paraformaldehyde (pH 9.5) instead of sodium-phosphate-buffered 4% paraformaldehyde buffer (pH 7.4).

### Immunohistochemistry

Perfused brains were sectioned (25 μm) on a freezing sliding microtome. pSTAT3 immunohistochemistry was performed as previously described^31^ on every sixth section, using a rabbit anti-pSTAT3 antibody (Cell Signaling Technology, Inc., Danvers, MA, USA; catalogue no. 9145, 1:250). pSTAT3 immunoreactive positive cells were counted bilaterally under a light microscope by an investigator blind to the treatment groups, through arcuate nucleus, dorsomedial nucleus (DMH) and ventromedial nucleus (VMH) of the hypothalamus. pSTAT3-positive cells were expressed as an average per area on each side of the third ventricle. Regions were analysed anatomically matched across all animals with three sections being analysed per animal.

For GFAP immunohistochemistry, tissue was rinsed with a phosphate-buffered saline (PBS)/0.1% Triton X-100, followed by blocking in 1% hydrogen peroxide in tris-buffered saline (TBS) for 10 min, rinsing in TBS/0.05% tween for 5 min, and blocking in 0.2% bovine serum diluted in PBS/0.1% Triton X-100 for 20 min. Tissue was incubated for 1 h at room temperature with a rabbit anti-GFAP antibody (Dako Denmark A/S, Glostrup, Denmark; catalogue no. Z0334, 1:1000). Following primary incubation, tissue was rinsed before 1 h of incubation at room temperature with an anti-rabbit horseradish peroxidase antibody (Jackson ImmunoResearch Laboratories, Inc., West Grove, PA, USA; catalogue no. 711-035-152, 1:500). The final step after the tissue was rinsed again was incubation in a nickel sulphate DAB solution in sodium acetate. GFAP immunoreactive positive cells are expressed on astrocytes and were quantified as through ARC of the hypothalamus. The scanned images were analysed using Visiomorph 4.3.1.0 (Visiopharm, Hoersholm, Denmark). For analysis, a bayesian classifier was trained with positive and negative GFAP-areas blindly to the treatment groups. GFAP-positive cells were expressed as an average percent per area on one side of the third ventricle for each animal. Regions were analysed anatomically matched across all animals with three sections being analysed per animal distributed from −3.12 to −3.48 mm from the bregma.

### Statistics

Data obtained from the experiment was primarily analysed in the statistical program R version 2.15.3 and graphs were created in GraphPad Prism version 6.0.

The statistical analysis used for data obtained from the experiment was performed by fitting data as mixed linear models (lme). Data were analysed either as one-, two- or three-factor study designs (ANOVA). The fixed factors were ‘Maternal diet (DietM)', ‘offspring diet (DietO)' and ‘Dosis (Dos)' (used for the measurement of the leptin sensitivity). Furthermore, dam gestation ‘DG' (refers to the dam from which the offspring was born) was used as a random factor. All variables were visually assessed and statistical tested by ‘Shapiro–Wilk normality test' for normal distribution. Also, homogeneity of variance were assessed by visual inspection of residual plot and tested through ‘Bartlett test of homogeneity of variances'. The replicate of the statistical analysis was referred to individual offspring in each group. Data are presented as means±s.e.m. A *P*-value of 0.05 or less was considered statistically significant.

## Results

### Pregnant and lactating mothers

Maternal body weight and percentage of body fat mass was not affected by the S diet during gestation. However, by the end of lactation percentage of body fat mass was increased in S-fed mothers (*P*<0.01) but there were no differences in body weight. No effect was observed on blood glucose, plasma insulin, TG and FFA levels during gestation and lactation (data not shown). Therefore, the possible changes in foetus development were a direct effect of the maternal intake of the S diet. Furthermore, pregnancy length, litter size and offspring birth weight did not differ between C- and S-diet-fed mothers (data not shown).

### Offspring (birth to weaning)

Birth weight was not affected by the maternal S diet (*P*=0.24; data not shown). At day 1 after birth, no significant differences were observed in blood glucose or plasma leptin (*P*=0.11, *P*=0.60, respectively); however, plasma insulin was increased in offspring born to mothers fed the S diet (*P*<0.05; Table 1). At weaning (3 weeks of age), blood glucose was unaffected in S offspring (*P*=0.09) despite a significant decrease in plasma insulin (*P*<0.01). Furthermore, 3-week-old S offspring had increased plasma leptin (*P*<0.001) and plasma ghrelin (*P*<0.05; Table 1) compared with C offspring. Messenger RNA (mRNA) levels were increased for anorexigenic neuropeptide CART (*P*<0.05) and close to significantly increased for POMC (*P*=0.06) in offspring from S-fed mothers compared with offspring from C-fed mothers, whereas no effect of the maternal diet was observed for mRNA levels of hypothalamic orexigenic neuropeptides NPY, AgRP, GALp and anorexigenic neuropeptide MC4R.

### Offspring (11–12 weeks)

**Figure 2:**
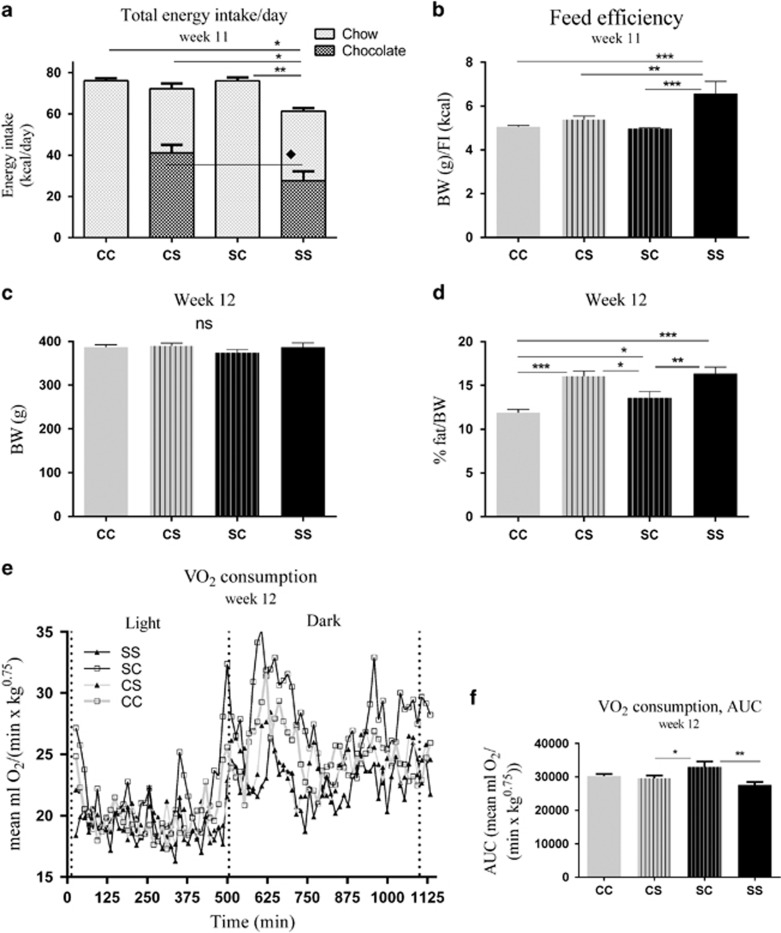
Body weight, body fat mass and energy metabolism in adolescent offspring (week 11–12). (**a**) Energy intake, (**b**) feed efficiency, (**c**) body weight, (**d**) body fat mass, (**e**, **f**) VO_2_ consumption. Data are expressed as means±s.e.m., *n*=8–10, analysed by mixed linear models (lme), unpaired *t*-test in R. (*P<0.05, **P<0.01, ***P<0.001). C, chow diet; CC, maternal C–post-weaning C; CS, maternal C–post-weaning S; S, chow diet supplemented with chocolate and soft drink *ad libitum*; SC, maternal S–post-weaning C; SS, maternal S–post-weaning S.

At 11 weeks of age, total energy intake was significantly decreased in SS compared with all the other groups: CC (*P*<0.05), CS (*P*<0.05), SC (*P*<0.01). Chocolate intake was furthermore slightly decreased in the offspring from S-fed mothers (SS) compared with those from C-fed mothers (CS; *P*<0.05; Figure 2a), which was primarily ascribed to differences in intake during the dark period. Decreased energy intake in SS was associated with an increased feed efficiency (body weight (g)/energy intake (kcal)) compared with CC (*P*<0.001), CS (*P*<0.01) and SC (*P*<0.001; Figure 2b). From weaning to 12 weeks of age, body weight was not significantly different between the groups (Figure 2c; only body weight at 12 weeks of age is shown). However, body fat mass was increased in CS and SS offspring at week 12 compared with CC (*P*<0.001) and SC (*P*<0.05, *P*<0.01; Figure 2d), and among the C-fed offspring, those born to S-fed mothers (SC) had higher amount of body fat compared with those born to chow-fed mothers (CC; *P*<0.05). Furthermore, the energy expenditure expressed as VO_2_ consumption was increased in SC compared to SS (*P*<0.01), CS (*P*<0.05) and CC (*P*=0.07; Figures 2e and f).

The mRNA levels of hypothalamic CART was significant reduced in SC offspring compared with SS offspring (*P*<0.05), however no other significant differences were observed in mRNA expression for neuropeptides in offspring aged 12 weeks.

### Adult offspring (aged 23–26 weeks)

**Figure 3:**
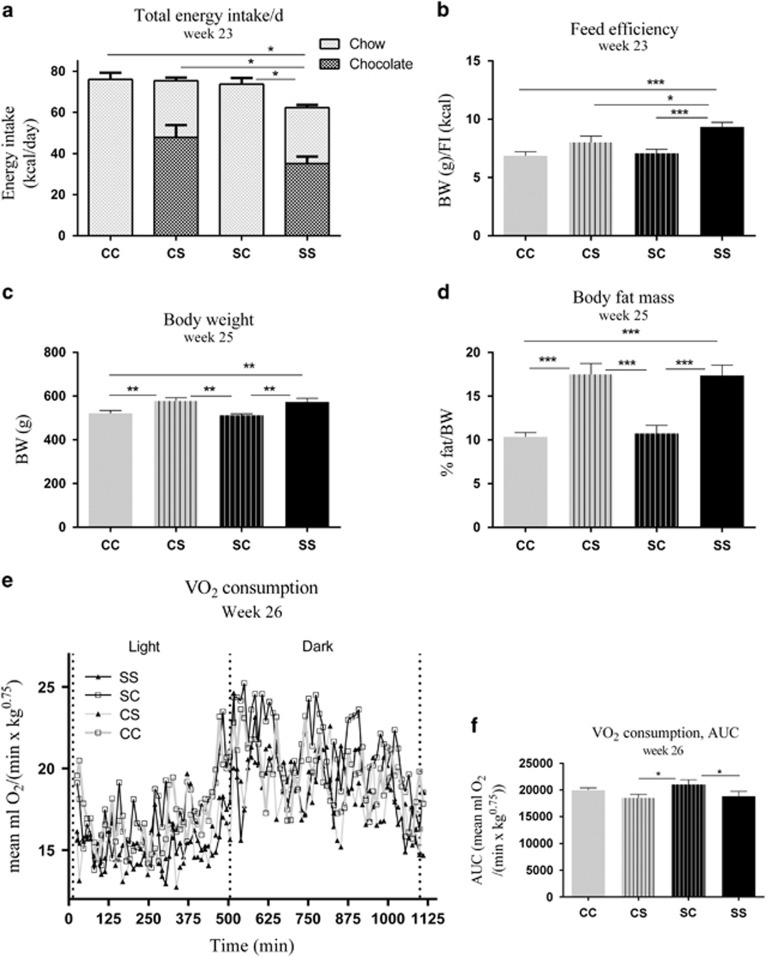
Body weight, body fat mass and energy metabolism in adult offspring (week 23–26). (**a**) Energy intake, (**b**) feed efficiency (**c**) body weight, (**d**) body fat mass, (**e**, **f**) VO_2_ consumption. Data are expressed as means±s.e.m., *n*=8–10, analysed by mixed linear models (lme), unpaired *t*-test in R. (*P<0.05, **P<0.01, ***P<0.001). AUC, area under the curve; C, chow diet; CC, maternal C–post-weaning C; CS, maternal C–post-weaning S; S, chow diet supplemented with chocolate and soft drink *ad libitum*; SC, maternal S–post-weaning C; SS, maternal S–post-weaning S.

At 23 weeks of age, SS had a lower total daily energy intake compared with CC (*P*<0.05), CS (*P*<0.05) and SC (*P*<0.05), resulting mainly from differences in chow intake, as chocolate intake did not differ between SS and CS (*P*=0.15) offspring (Figure 3a). Hence, feed efficiency was increased in SS compared with CS (*P*<0.05), CC (*P*<0.001) and SC (*P*<0.001; Figure 3b). From 13 weeks of age, CS and SS offspring had a higher body weight compared with SC offspring (*P*<0.05, *P*<0.01), and from 18 weeks of age, the body weight of CS and SS offspring were also higher than in CC offspring (*P*<0.01). This continued until 25 weeks of age (*P*<0.01; Figure 3c; only body weight at 25 weeks of age is shown). Furthermore, the postnatal S diet continued to result in increased body fat mass in CS and SS at week 25 compared with SC (*P*<0.001) and CC (*P*<0.001), while SC offspring normalized their body fat mass to the same level as in CC offspring (Figure 3d). At 25 weeks of age, SS and CS offspring had a lower VO_2_ consumption compared with SC (*P*<0.05, *P*<0.05, respectively; Figures 3e and f).

**Figure 4:**
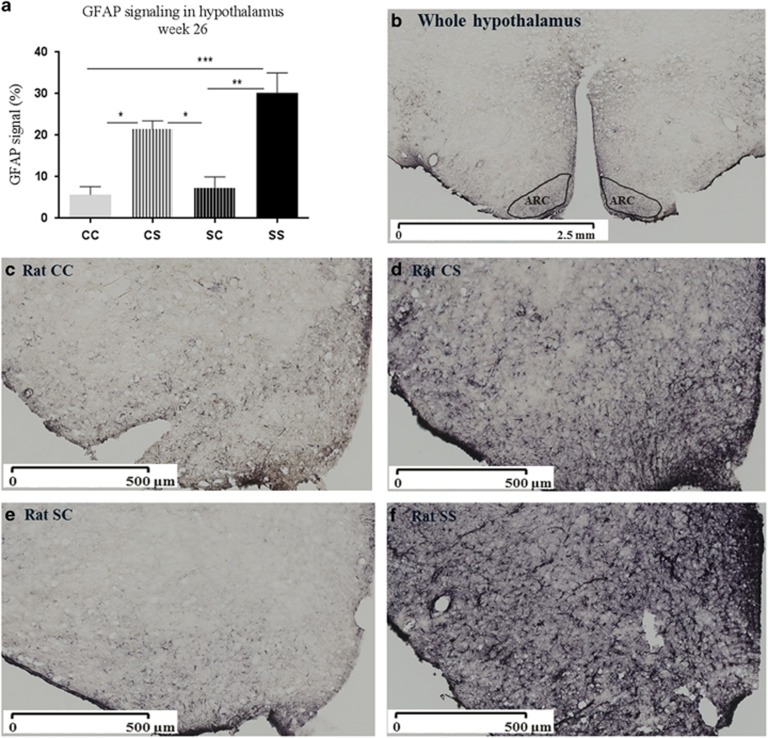
Hypothalamic astrocytes in adult offspring aged 26 weeks. Representative images of astrocytes were identified by immunohistochemical detection of GFAP protein. (**a**) Quantification of GFAP signal in the hypothalamus, (**b**) drawing of ARC in hypothalmus used for analysis, (**c**) astrocytes in CC offspring, (**d**) astrocytes in CS offspring, (**e**) astrocytes in SC offspring, (**f**) astrocytes in SS offspring. The images were scanned in × 20 magnification using a Hamamatsu Nanozoomer 2.0 HT (Hamamatsu Photonics K.K., Japan). Scale bar, 500 μm. Data are expressed as means±s.e.m., *n*=3–4, analysed by mixed linear models (lme), unpaired *t*-test in R. (*P<0.05, **P<0.01, ***P<0.001). CC, maternal chow–post-weaning chow; CS, maternal chow–post-weaning S; SC, maternal S–post-weaning chow; SS, maternal S–post-weaning S; C, chow diet; S, chow diet supplemented with chocolate and soft drink *ad libitum*.

At 24 weeks of age, a leptin challenge was performed in offspring exposed to post-weaning S diet (SS and CS). Food intake was measured at 4, 12 and 24 h after a single dose of leptin was administered i.p. Figure 4a shows that offspring irrespectively of the maternal diet responded to the injected leptin by reducing food intake 4 h after the injection compared with the saline injection (*p*<0.0001). No differences were observed in total energy intake 12 and 24 h after the leptin injection (data not shown).

Fasting plasma leptin levels was in offspring after an overnight fast at week 26. There was an overall significant effect of the postnatal S diet (*P*=0.03), but no significant differences between the groups were found (SS vs. CC, *P*=0.11 & CS vs. CC, *P*=0.07; Figure 4b). Fasting hypothalamic pSTAT3 positive cells in ARC, VMH and DMH were also measured. The combination of maternal and postnatal exposure to the S diet induced a reduction in fasting pSTAT3 activation in ARC of SS offspring compared with the three other groups (*P*<0.05; Figures 4c–g). No significant difference in pSTAT3 positive cells was observed in VMH and DMH (data not shown).

**Figure 5:**
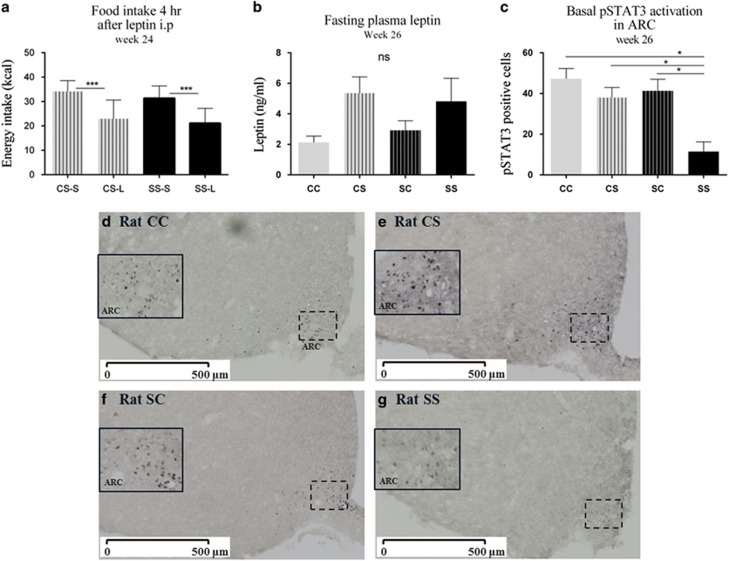
Leptin responsiveness in adult offspring (week 24–26). (**a**) Leptin challenge, energy intake, (**b**) fasting plasma leptin, (**c**) quantification of fasting pSTAT3 activation in ARC of the hypothalamus, (**d**) fasting pSTAT3 activation in CC offspring, (**e**) fasting pSTAT3 activation in CS offspring, (**f**) fasting pSTAT3 activation in SC offspring, (**g**) fasting pSTAT3 activation in SS offspring. Representative images of pSTAT3 nucleus were identified by immunohistochemical detection. The images were scanned in × 20 magnification using a Hamamatsu Nanozoomer 2.0 HT (Hamamatsu Photonics K.K., Japan). The full-line boxes are magnification of the stippled boxes illustrating the ARC. Data are expressed as means±s.e.m., *n*=4–10, analysed by mixed linear models (lme), unpaired *t*-test in R. (*P<0.05, **P<0.01, ***P<0.001). C, chow diet; CC, maternal chow–post-weaning chow; CS, maternal chow–post-weaning S; CS-S/CS-L, maternal chow–post-weaning S with saline (S) or leptin (L) injection; S, chow diet supplemented with chocolate and soft drink *ad libitum*; SS-S/SS-L, maternal S–post-weaning S with saline (S) or leptin (L) injection; SC, maternal S–post-weaning chow; SS, maternal S–post-weaning S.

The quantification of GFAP positive cells in the hypothalamus showed that postnatal S diet increased GFAP positive cells in CS offspring and SS offspring compared with CC (*P*<0.05, *P*<0.001) and SC offspring (*P*<0.01, *P*<0.001; Figure 5).

## Discussion

In the present study, we demonstrated that SS offspring, born and nursed by mothers fed the chocolate and soft drink supplementation (S) diet and subsequently exposed to the S diet throughout post-weaning life, became obese despite a lower energy intake compared with all other groups. This was due to the increased feed efficiency (body weight (g)/food intake (kcal)) and reduced leptin signalling in ARC as indicated by reduced fasting pSTAT3 activity. These findings suggest that prenatal exposure to the S diet predisposed for long-term changes in neuronal function of ARC with implications for the regulation of energy balance, and this became phenotypically manifested only upon subsequent exposure to the S diet also in postnatal life. On the other hand, adult offspring exposed to maternal S and fed postnatal chow after weaning (SC) had comparable body weight, fat mass and leptin sensitivity to that of the control group. Thus, the changes induced by the maternal S diet could apparently be reversed by exposure to a healthy nutrient after weaning which is in agreement with previous findings.^31, 32, 33, 34^

Certainly, neuroendocrine function appeared to be altered in adult SS offspring, where the unique finding was that these offspring became obese despite decreased energy intake co-existing with increased feed efficiency and reduced fasting leptin signalling. This deviates from most other rodent studies, where offspring from obese mothers or from HFD-fed mothers became obese associated with increased plasma leptin, increased energy intake^35^ as well as leptin resistance.^23, 25^ The primary role of leptin in the regulation of energy homoeostasis is to suppress food intake and increase energy expenditure.^1, 2, 3^ However, recent studies have suggested that leptin resistance may develop selectively in some pathways while sensitivity is preserved in others, indicating differential regulation in distinct tissues/brain areas. For instance, in diet-induced obese mice, leptin acted to stimulate sympathetic innervation regulating blood pressure, but failed to regulate energy homoeostasis.^36^ In the present study, both SS and CS offspring had slightly increased fasting plasma leptin levels associated with reduced energy expenditure, indicating reduced response of leptin to suppress energy expenditure. In addition, no indications of leptin resistance within appetite regulation were observed as both CS and SS offspring responded well to an intraperitoneal injection of leptin by reducing energy intake. SS offspring were furthermore able to lower energy intake during normal circadian feeding despite reduced pSTAT3 activity in ARC, indicating that neurons regulating appetite were more sensitive to the effect of leptin than neurons regulating energy expenditure. pSTAT3 evaluation of other areas of the hypothalamus known to express LEPR such as the VMH and DMH showed insignificant low fasting pSTAT3 activation, indicating the ARC was the most prominent area of leptin signalling during fasting.^37^ As SS offspring became obese despite a lower energy intake and reduced leptin signalling in ARC, the findings suggest changes in neuronal function of ARC with implications for the regulation of energy balance, and lends support to the possibility that leptin sensitivity in different metabolic systems within the same organ (ARC of the hypothalamus) can be differentially expressed. From that perspective, it could have been interesting to investigate energy expenditure in a response to injection of leptin as well as molecular examination of neurons regulating energy balance in the ARC. Previously, it has been found that mice selectively deficient for LEPR in POMC neurons developed modest obesity that was associated with reduced energy expenditure independent of changes in food intake.^38^ Therefore, the reduced effect of leptin in the regulation of energy expenditure in the present study could be related to low expression of LEPR or deficiency in leptin down-stream signalling on POMC neurons important for regulation of energy expenditure.^38^

The observed hypothalamic astrogliosis in ARC could potentially also affect neurons responsible for regulating leptin signalling,^4, 5, 6^ and this could be more pronounced in neurons regulating energy expenditure. Astrogliosis is accompanied by increased expression of inflammatory agents^7, 39, 40, 41^ and modulate the inflammatory response in neurons that damage the neurons^41^ and may also impair leptin signalling in hypothalamic neurons.^4, 5, 6^ From the study results, astrogliosis as a response to neuronal damage was observed as a consequence of long-term S feeding in both CS and SS offspring, consisting with long-term HFD feeding in rodents by Thaler *et al.*^7^ According to Thaler *et al.*, the astrogliosis was associated with neuronal damage within the hypothalamus causing dysregulation of energy balance contributing to the obese phenotype.^7^ In the present study, astrogliosis was not significantly associated with the reduced leptin signalling ARC in SS offspring, although there was a tendency of increased astrogliosis in SS offspring compared with CS offspring. In addition, it could have been valuable if the paraventricular nucleus (PVN), VMH, DMH and the lateral hypothalamus (LHA) were investigated as these areas also are important for regulation of energy balance.^42^ However, on the existing slides with ARC, GFAP positive cells appear to be increased in VMH (not stereological represented), suggesting that astrogliosis also were present in VMH.

The manifested obese phenotype in SS offspring may likely be traced back to the pre- and early postnatal life as the mothers intake of HFD are shown to affect the offspring development and predispose to metabolic diseases later in life.^15, 16, 17^ The rat brain starts to develop during mid-gestation,^15, 18, 43^ while hypothalamic neuron circuits mainly develop after birth and within the first weeks of life.^18, 44, 45^ Changes in neurotrophic factors such as leptin, insulin and ghrelin during early postnatal life have been shown to induce hypothalamic changes in structure and neuronal function in the offspring.^15, 18, 46^ In the present study, feeding the S diet to mothers during gestation and lactation resulted in increased circulating levels of insulin in their offspring at day 1, as well as increased levels of leptin and ghrelin and decreased levels of insulin at week 3 after birth. S offspring had increased mRNA level of CART (anorectic peptide) in hypothalamus that likely was induced by increased leptin.^47^ Expression levels of other studied neuropeptides were not affected by the neurotrophic hormones; however, this does not rule out that other hypothalamic neuroendocrine functions could be altered later in life.^15, 18^ In addition, previously histological analysis of neonatal insulin-treated rats revealed axonal projection formation was reduced while offspring with hypoinsulinemia had reduced immunoreactive cells of GALp and NPY in ARC.^48^ Also, leptin deficiency or increased leptin exposure in early postnatal life have been shown to induce disruption in neuronal circuit formation in ARC by reducing axonal projection of both AgRP/NPY and POMC/CART neurons to PVN^26^ or by increasing NPY expression in PVN and CART.^49^ Hereby, changes in neutrophic factors during early life have been found to disrupt neuronal development co-existing with development to abnormal regulation of energy balance leading to obesity^26, 28^ which likely could be the circumstance in this study. At 12 weeks of age, SS offspring had normalized the CART mRNA levels compared with the controls while SC had reduced CART levels compared to SS. We do not have any good explanation for this result. However, it did not show to have any significant impact in the regulation food intake in SC offspring. Prospectively, histological analysis of hypothalamic neuronal formation in early life would be preferred as a supplement to qPCR to investigate hypothalamic development and its neuroendocrine function in controlling energy balance.

Finally, we also observed that S offspring at 3 weeks of age had reduced insulin levels and normal glucose levels, indicating a less demand for insulin due to compensatory increased insulin sensitivity. This is consistent with our previous study.^29^ As the hypothalamus also regulates the peripheral glucose homoeostasis;^50^ the results hereby indicated that hypothalamic neurons responsible for regulating glucose homoeostasis were not affected by the maternal S diet neither at weaning or adulthood at 25 weeks of age (M Kjaergaard, C Nilsson, MO Nielsen, KL Grove and K Raun, in preparation). This conclusion was confirmed by White *et al.*^51^ showing that glucose homoeostasis was unaffected by maternal obesity and offspring diet in 20 weeks old offspring despite early changes of the insulin sensitivity. Thus, both studies indicated that longer-term evaluation was an important aspect when investigating effects of maternal obesity and diet on glucose metabolism.

In conclusion, as expected adult offspring fed the S diet post weaning became obese associated with reduced energy expenditure and hypothalamic astrogliosis irrespective of maternal diet. Interesting, maternal intake of chocolate and soft drink (S diet) had long-term adverse consequences for the metabolic phenotype in the offspring when they continued to ingest the S diet themselves in postnatal life. These SS offspring developed obesity due to increased feed efficiency despite a reduced energy intake associating with reduced leptin signalling in ARC as indicated by reduced fasting pSTAT3 activity. Our data suggest that SS offspring must have been more responsive to the depressive impact of leptin on appetite regulation than to the stimulatory impact on energy expenditure, which indicates differential programming of leptin sensitivity in ARC. Future studies are needed to distinguish whether reduced fasting leptin sensitivity in SS offspring is differentially located in different neurons of the hypothalamus.

## Figures and Tables

**Table 1:** Plasma metabolites in early postnatal life at day 1 and week 3 of age (acting as neurothrophic factors) and mRNA levels of neuropeptides in the hypothalamus at week 3 and 12 of offspring

*Plasma metabolites*	*Glucose (mmol l^−1^)*	*Insulin*^a^ *(pmol l^−1^)*	*Leptin (ng ml^−1^)*	*Ghrelin (pg ml^−1^)*
*Day 1*				
C	4.65±0.1	192±34	2.6±0.6	—
S	4.92±0.1	307±42*	2.9±0.9	—
				
*Week 3*				
C	6.9±0.1	73.6±5.6	1.5±0.1	28.4±2.9
S	7.4±0.2	53.9±2.0^**^	3.2±0.2^***^	56.7±9.3*

*mRNA neruropeptides*	*NPY*	*AgRP*	*GALp*	*POMC*	*CART*	*MC4R*
*Week 3*						
C	2.27±0.4	3.12±0.5	0.56±0.1	1.23±0.3	0.73±0.1	4.6±0.9
S	6.54±0.6	4.72±0.7	0.79±0.1	2.80±0.9	1.47±0.2*	6.3±1.2
						
*Week 12*						
CC	0.54±0.1	0.39±0.1	1.19±0.1	0.77±0.2	1.46±0.1	1.18±0.1
CS	0.70±0.1	0.33±0.1	1.54±0.2	0.93±0.3	1.36±0.3	0.77±0.2
SC	0.41±0.1	0.35±0.1	0.79±0.1	0.51±0.1	0.53±0.1*,b	0.28±0.1
SS	0.68±0.1	0.24±0.1	1.47±0.4	1.03±0.4	1.55±0.2	0.57±0.1

Abbreviations: CC, maternal chow–post-weaning chow; CS, maternal chow–post-weaning S; S, chocolate and soft drink supplement; SC, maternal S–post-weaning chow; SS, maternal S–post-weaning S.

a Plasma insulin at day 2.

b Indicates significant difference to SS.

Relative mRNA levels of each neuropeptide are presented as a percentage of the mRNA level of 18 S ribosomal RNA. Data are expressed as means±s.e.m., *n*=8–10, analysed by unpaired *t*-test and mixed linear models (lme) in R. (*P<0.05, **P<0.01, ***P<0.001).
